# Predictors for Abundance of Host Flea and Floor Flea in Households of Villages with Endemic Commensal Rodent Plague, Yunnan Province, China

**DOI:** 10.1371/journal.pntd.0000997

**Published:** 2011-03-29

**Authors:** Jia-Xiang Yin, Alan Geater, Virasakdi Chongsuvivatwong, Xing-Qi Dong, Chun-Hong Du, You-Hong Zhong

**Affiliations:** 1 Yunnan Institute of Endemic Disease Control and Prevention, Dali City, Yunnan Province, People's Republic of China; 2 Epidemiology Unit, Faculty of Medicine, Prince of Songkla University, Hat Yai, Thailand; Swiss Tropical Institute, Switzerland

## Abstract

**Background:**

From 1990 to 2006, fifty-five natural villages experienced at least one plague epidemic in Lianghe County, Yunnan Province, China. This study is aimed to document flea abundance and identify predictors in households of villages with endemic commensal rodent plague in Lianghe County.

**Methods:**

Trappings were used to collect fleas and interviews were conducted to gather demography, environmental factors, and other relevant information. Multivariate hurdle negative binomial model was applied to identify predictors for flea abundance.

**Results:**

A total of 344 fleas were collected on 101 small mammals (94 *Rattus flavipectus* and 7 *Suncus murinus*). *R. flavipectus* had higher flea prevalence and abundance than *S. murinus*, but the flea intensities did not differ significantly. A total of 315 floor fleas were captured in 104 households. *Xenopsylla cheopis* and *Ctenocephalides felis felis* were the predominant flea species on the host and the floor flea, respectively. The presence of small mammal faeces and *R. flavipectus* increased host flea prevalence odds 2.9- and 10-fold, respectively. Keeping a dog in the house increased floor flea prevalence odds 2-fold. Keeping cattle increased floor flea intensity by 153%. Villages with over 80% of houses raising chickens had increased prevalence odds and intensity of floor flea about 2.9- and 11.6-fold, respectively. The prevalence and intensity of floor flea in brick and wood houses were decreased by 60% and 90%, respectively. Flea prevalences of host and floor flea in the households that were adjacent to other houses were increased 7.4- and 2.2-fold, respectively. Houses with a paddy nearby decreased host flea intensity by 53%, while houses with an outside toilet increased host flea intensity by 125%.

**Conclusion:**

Rodent control alone may not be sufficient to control plague risk in these areas. In order to have successful results, plague control programs should pay attention to ecological and hygiene factors that influence flea populations.

## Author Summary

Yunnan province is located in southwest China. Plague is still a huge threat to the health of local people in Yunnan where plague epidemics had the most serious impacts than other provinces in China. The risk of plague outbreak is driven by rodent and flea populations. Our research team is conducting a study to identify predictors for abundance of host and floor fleas in households of villages with endemic commensal rodent plague in Yunnan province. The results of this study will contribute to control host and floor flea populations, and therefore to prevent and control plague outbreaks in these areas.

## Introduction

In China, animal plague has been reported almost every year and human plague outbreak occasionally occurred [1]; [2]. Of 11 geographical foci of plague, commensal rodent plague foci have the highest reported human cases in southern and south-western China. Human cases in Yunnan province accounted for around 60% of total plague cases in China during the period from 1986 to 2005 [3].

Although recent studies reported that rodent and flea abundance fail to predict a sylvatic plague epizootic [4]; [5], the size of small mammal population and the abundance of the flea on these hosts are important indicators for plague control in many systems [2]; [6]–[10]. In the commensal rodent plague areas of China, it was demonstrated that the density of host and floor flea had a positive relationship with rodent plague epidemic [11]. As floor flea is believed to have a high potential to attack human, floor flea density measurements have been routinely taken for plague control in China. However, the correlation between host and floor flea abundance and whether the two types of flea share the same environmental predictors have not been reported.

Among abiotic factors, the ambient temperature and relative humidity are the two most important factors influencing the birth and death rate of flea [12]; [13]. Human behaviour also affects the population size of flea in households of villages with endemic commensal rodent plague. To improve plague prevention and control programs in these areas, a better understanding of predictors for abundance of host and floor fleas in households is needed. Our study consisted of a small mammal part and a flea part. The first part has been presented [14]. This report focus on documenting the abundance of host and floor flea and on identifying predictors in households of villages with endemic commensal rodent plague.

## Methods

### Ethics statement

This study was approved by the institutional research commissions of Yunnan Institute of Endemic Diseases Control and Prevention (China) and the Ethics Committee of the Faculty of Medicine, Prince of Songkla University (Thailand). Written informed consent (in Chinese) was obtained from all participants of the study. All animal work was conducted with ethical approval from the Ethics Committee, Faculty of Medicine, Prince of Songkla University (SUB.EC 51/354-001). According to “Chinese Regulations for the Administration of Experimental Animals (modified in 2004)” and “Yunnan Provincial Regulations for the Administration of Experimental Animals (established in 2007)”, all captured small mammals (with possibility of carrying *Yersinia pestis*, the aetiological agent of plague) were burned after collecting fleas.

### Study design

A cross-sectional study was applied. Field investigations were carried out in Lianghe county, Dehong prefecture, Yunnan province, China, from August to September 2007.

### Study setting

Lianghe County is one of 5 counties bordering Myanmar in Dehong prefecture. In 2002, the total population was about 160,000 (89% of them farmers). Ethnic groups include Han, Dai, A Chang, Jing Po, De Ang and others. The minority populations account for about 33% of the total population in this county. The average annual temperature is 18.3°C, average annual rainfall is 1396.2 mm, and average annual sunshine is 2385.5 hours. Economy mainly relies on agriculture. The average net income of farmers was 816 RMB (about US$100) annually [15].

In 1990, rodent plague re-emerged in this county after a 33-year quiescent period. From 1990 to 2006, among 381 villages of Lianghe County, 55 experienced at least one plague epidemic. Six villages had human and rodent plague and 49 villages had rodent plague only.

### Study villages and households sampling

Thirty-four villages experienced at least one rodent plague epidemic in Lianghe County in the six years from January 2001 to December 2006. Thirty of these were selected as study villages. Four were excluded because of access difficulties. Of these 30 villages, the number of villages experiencing 1, 2, 3 and 4 epidemics in the past 6 years was 17, 9, 2 and 2, respectively.

A list of all households was obtained from the local village administration for the 30 villages. In eleven unusually large villages, the largest subdivision was taken as the representative study unit. Households of each village were given a unique code, and 20 households per village were randomly selected using computer-generated random numbers.

### Survey for determinants of flea abundance

Village- and household-level data were collected using questionnaire and observation checklist. At the village level, a face-to-face questionnaire-based interview was conducted with a leader of the village to obtain information on the main source of economy, number of households and persons, major ethnic group, having domestic animals, and past rat and flea control. The observation checklist covered topography and presence/location of rubbish areas in the villages.

At the household level, the head of the household or spouse were interviewed face-to-face using a questionnaire covering details of ethnic group, presence of domestic animals in the house, recent experience of seeing any small mammal (SM) and/or its faeces in the house, and having a rat problem. A household observation checklist covering the type of house construction, the surroundings of the house, the presence of SM faeces, crops grown near house (within 50 meters) was also used.

Data was collected by three trained interviewers from Yunnan Institute of Endemic Disease Control and Prevention (YIEDC). Each potential participant was given a clear explanation of the research purpose and asked to sign an informed consent form before any data was collected.

### Small mammal trapping and flea collection

SM trapping was carried out by placing 5 live-traps (20×12×9 cm) per house on two consecutive nights. SMs captured were identified to species in the field according to their morphological features. Cages with captured SMs were put into plastic bags and brought to the laboratory for collecting fleas.

After anesthetizing the SMs with aether, their fur was brushed until all fleas were recovered. The collected fleas were placed in labelled vials containing 75% ethanol. The fleas from each SM were preserved in one vial.

Floor fleas, defined as a population as yet unfed or dissociated from host and seeking for a new host, were trapped using self-made sticky paper (A4 size). Four rooms of each household were selected for placing 20 sticky papers; five papers per room (4 at the corners and 1 in the centre) were placed in the afternoon and collected in the next morning. The trapped fleas were preserved in labelled vials containing 75% ethanol and subsequently identified to species under a light microscope by an entomologist of YIEDC.

### Statistical analysis

Data was coded and computerized with EpiData software [16] and analyzed using R software [17]. Host-, household- and village-level information were summarized using descriptive statistics.

The following international definitions for various host flea indicators were adopted [18]: Flea prevalence  =  (number of hosts infested with flea/total number of captured hosts) * 100; Flea intensity  =  total number of fleas/number of hosts infested with flea; Average flea abundance  =  total number of fleas/total number of captured hosts. For the floor fleas, the commonly used Chinese definition of general floor flea index (number of floor flea captured/number of sticky papers) was adopted [19]. Furthermore, floor flea prevalence per house, floor flea intensity per infested house and average floor flea abundance were also adopted. The similar statistical approaches were used for both host and floor fleas.

Flea prevalence by host species was compared using chi square test. Differences on flea intensity and flea abundance were tested using rank sum test. The correlation of co-occurring flea species on *R. flavipectus* and of two major floor flea species in the houses were explored using Spearman rank correlation coefficient. The association between the prevalence of floor fleas and of SMs/host fleas in the same household were explored using chi square test.

Hurdle negative binomial (HNB) regression model was applied to account for the current cross-section data set exhibits over-dispersion and excess zeros. The model is a two-component model: one is logistic model fitting zero vs. larger counts, the other is negative binominal model fitting positive counts [20]. For univariate and multivariate analysis, predictors for flea prevalence (logistic regression component) with predictors for flea intensity (negative binomial regression component) were integrated in HNB regression models. Thus, the model will identify factors affecting two components of the flea abundance (average flea abundance  =  flea prevalence * flea intensity). The first set of predictors predicts whether the host or the house would be infested by any flea. The second set of predictors predicts the intensity of fleas among infested hosts or houses. Independent variables with p<0.2 were included in subsequent corresponding part of the prototype multilevel HNB regression model. The final models were refined using backward elimination to reduce independent variable predicting neither the prevalence nor the intensity (using p<0.05 as the criterion for statistical significance). Coefficients and 95% CI of the logistic regression component were exponentiated to obtain prevalence odds ratios (OR). Similarly, those of the negative binomial (NB) component were exponentiated to yield intensity ratios (IR).

## Results

A total of 600 households from 30 villages with endemic commensal rodent plague were surveyed. *Rattus flavipectus* (133) and *Suncus murinus* (33) were trapped. Host fleas (range: 1–31 fleas per household) and floor fleas (range: 1–59 fleas per household) were collected in 75 and 104 households, respectively. Fifteen households had fleas from both host and floor. There was no relationship between the prevalences of host and floor fleas in the same household (chi square test, p  =  0.625). Sixty-eight households had *R. flavipectus* which infested by flea, while 7 households had at least 1 infested *S. murinus*.

The mean abundance, prevalence and intensity of host flea by the two SM species are shown Table 1. The general flea prevalence, flea intensity and average abundance were 60.8%, 3.41 and 2.07, respectively. The flea prevalence of *R. flavipectus* (70.7%) was significantly higher than that of *S. murinus* (21.2%) (chi square test, p < 0.001), but the flea intensity of *R. flavipectus* and *S. murinus* was not significantly different (rank sum test, p  =  0.082). The flea abundance of *R. flavipectus* (2.48 fleas per host) was significantly higher than that of *S. murinus* (0.42 fleas per host) (rank sum test, p < 0.001). In summary, the risk of flea infestation was higher for *R. flavipectus* than that for *S. murinus*. However, there was no evidence that once infested, the number of fleas per host on these 2 SM species were different.

**Table 1:** Flea prevalence, flea intensity and average flea abundance by two small mammal species.

Variable	*R. flavipectus*	*S. murinus*	Total	P value
Number of SMs examined	133	33	166	
Number of SMs infested	94	7	101	
Number of fleas	330	14	344	
Flea prevalence (%)	70.7	21.2	60.8	<0.001 a
Flea intensity (SD)	3.51 (3.88)	2.00 (2.24)	3.41 (3.81)	0.082 b
Flea abundance (SD)	2.48 (3.63)	0.42 (1.28)	2.07 (3.40)	<0.001 b

a P value from Chi square test.

b P value from rank sum test.

Flea source, numbers, species, and sex are shown in Table 2. *Xenopsylla. cheopis* was the dominant flea species on both species of SM., while *Leptopsylla segnis* was founded only on *R. flavipectus*. The numbers of *X. cheopis* and *L. segnis* were not correlated on *R. flavipectus* (Spearman rank correlation, ρ  =  0.09, p  =  0.303).

**Table 2:** Distribution of flea species and sex by flea source.

Flea source	*X. cheopis*		*P. irritans*		*L. segnis*		*C. felis felis*		Total
	M*	F*	M	F	M	F	M	F	
*R. flavipectus*	127	155			19	29			330
*S. murinus*	6	8							14
Floor	59	44	2	4		1	135	70	315
Total	192	207	2	4	19	30	135	70	659

*M  =  male F  =  female.

A total of 12,000 sticky papers was placed on floors and 11,888 (99.1%) were retrieved. A total of 315 fleas were recovered from these sticky papers. General flea index on floor (mean number of fleas per sticky paper) was 0.026 (315/11888). Floor flea prevalence (proportion of all houses that had floor fleas) was 17.3% (104/600), floor flea intensity was 3.03 fleas per infested house (315/104) and mean floor flea abundance was 0.53 fleas per house (315/600). Flea species on floors included *Ctenocephalides felis felis* (65.1%), *X. cheopis* (32.7%), *Pulex irritans* (1.9%) and *L. segnis* (0.3%). *C. felis felis* was the dominant flea on floors but was not found on either host. *P. irritans* was collected only on floors in small numbers. Both *X. cheopis* and *C. felis felis* were collected from floors of 15 houses and there was a weak positive association between the numbers of these two species of floor flea in the same house (ρ  =  0.19, p < 0.001).

There was no association between the capture of SMs and the collection of floor fleas in the same house (chi square test, p  =  0.904). A significant difference in *X. cheopis* flea overall sex ratio occurred between those on a host and those on the floor (chi square test, p  =  0.041). However, there was no difference in *X. cheopis* flea sex ratio between the 2 host species (Chi square test, p  =  0.908). These data, together with the different species composition, suggest that host fleas and floor fleas are largely distinct populations.

Table 3 shows the distribution and univariate analysis of number of fleas per host by host species and household variables. The odds of finding host fleas was higher on *R. flavipectus,* in houses where SM faeces were seen, had reported problems with small mammals, and were located adjacent to other houses. Flea intensity of host flea was higher in houses where vegetables were grown near houses and had outside toilets. There was no evidence that village-level variables influenced host flea abundance.

**Table 3:** Distribution and univariate analysis of the number of fleas per host by variables.

Variable	Number of fleas per host				Logistic part	Count part
	Mean (range)	0	1∼5	6∼25	P value	P value
Host species					<0.001	0.130
*S. murinus*	0.42 (0–7)	26	6	1		
*R. flavipectus*	2.48 (0–25)	39	78	16		
Seeing SM in house					0.870	0.126
No	1.62 (0–10)	16	23	3		
Yes	2.23 (0–25)	49	61	14		
Seeing SM faeces in house					<0.001	0.753
No	1.65 (0–25)	48	39	9		
Yes	2.66 (0–23)	17	45	8		
SM problem in house					<0.001	0.218
No	1.89 (0–25)	44	32	9		
Yes	2.26 (0–23)	21	52	8		
Surroundings - house					0.016	0.663
No	0.67 (0–4)	9	3	0		
Yes	2.18 (0–25)	56	81	17		
Vegetable grown near house					0.707	0.046
No	1.77 (0–25)	38	54	8		
Yes	2.53 (0–23)	27	30	9		
Paddy grown near house					0.614	0.081
No	2.26 (0–25)	50	66	15		
Yes	1.37 (0–7)	15	18	2		
Sugarcane grown near house					0.138	0.439
No	2.18 (0–25)	55	77	16		
Yes	1.17 (0–7)	10	7	1		
Location of toilet					0.215	0.016
No toilet	1.72 (0–11)	46	57	10		
Inside house	0.93 (0–4)	8	7	0		
Outside house	3.58 (0–25)	11	20	7		

Variables with a p-value of <0.2 in univariate analysis were entered into the corresponding part of a prototype multivariate hurdle negative binomial (HNB) regression model. Thus, the binomial part of the prototype multivariate HNB model had 5 variables, namely host species, seeing SM faeces, SM problem in house, surrounding-house, and sugarcane grown near house. The negative binomial (NB) part also had 5 variables, namely host species, seeing SM, vegetable grown near house, paddy grown near house, and location of toilet.

The distribution of household- and village-level variables and univariate analysis results for the number of floor fleas per household are shown in Table 4. Capture of floor fleas was more common among houses in villages in the mountains than those in basins, and in villages where a large proportion of households (>80%) raised chickens. Capture was also more common in houses that were constructed of earth and wood rather than brick and wood, raised chickens, kept a dog and/or were surrounded by other houses.

**Table 4:** Distribution of variables and univariate analysis results for number of floor fleas per household.

Variable	Number of floor fleas per house				Logistic part	Count part
	Mean (range)	0	1∼5	6∼59	P value	P value
**Village level:**						
Topography of village					<0.001	0.006
Mountain	0.97 (0–59)	164	49	7		
Basin among mountains	0.27 (0–6)	332	45	3		
Central waste areas in village					0.307	0.026
No	0.47 (0–20)	103	15	2		
Yes	0.76 (0–59)	393	79	8		
Major ethnic group					0.173	0.955
Han and other	0.63 (0–20)	143	32	5		
Dai	0.48 (0–59)	353	62	5		
Number of houses					0.281	0.013
≤80	0.43 (0–11)	243	53	4		
>80	0.62 (0–59)	253	41	6		
Houses raising chicken					0.002	0.004
≤80%	0.09 (0–2)	111	9	0		
>80%	0.63 (0–59)	385	85	10		
**Household level:**						
Keeping chicken					0.504	0.014
No	0.30 (0–6)	140	24	2		
Yes	0.61 (0–59)	356	70	8		
Keeping dog					0.008	0.231
No	0.37 (0–20)	322	48	5		
Yes	0.78 (0–59)	174	46	5		
Keeping pig					0.427	0.038
No	0.67 (0–59)	158	27	2		
Yes	0.46 (0–14)	338	67	8		
Keeping cattle					0.766	0.019
No	0.40 (0–14)	294	55	5		
Yes	0.71 (0–59)	202	39	5		
Seeing SM in house					0.770	0.014
No	0.71 (0–59)	184	33	4		
Yes	0.42 (0–20)	312	61	6		
SM capture					0.985	0.059
No	0.57 (0–59)	405	76	9		
Yes	0.35 (0–6)	91	18	1		
*R. flavipectus*					0.980	0.096
No	0.56 (0–59)	424	80	9		
Yes	0.35 (0–6)	72	14	1		
House construction					0.030	0.031
Earth and wood	0.58 (0–59)	428	88	10		
Brick and wood	0.11 (0–3)	68	6	0		
Surroundings - house					0.031	0.114
No	0.12 (0–3)	74	7	0		
Yes	0.58 (0–59)	422	87	10		

The numbers of floor flea in houses were higher in houses in mountain villages, in larger villages (>80 households), in villages where a large proportion (>80%) of households raised chickens, and in villages that had central rubbish areas. Floor flea numbers were also higher in houses that kept chickens, that kept cattle, and that were constructed of earth and wood rather than brick and wood. Floor flea numbers were lower in houses where rats were reported to be seen and that kept pigs.

Following the univariate analysis (Table 4), the 6 and 12 variables, respectively, that have shown some evidence of association in the binomial and count models were entered into the binomial part and count part of the prototype multivariate HNB model.

Table 5 shows the results of the final multivariate HNB regression model for number of fleas per host and number of floor fleas per household. *R. flavipectus* was more likely to be infested than *S. murinus*. Seeing small mammal faeces in the house and the house being located adjacent to other houses also increased the odds of small mammals been infested. Growing paddy near the house decreased, and having an outside toilet increased, the intensity of infestation among small mammals.

**Table 5:** Adjusted prevalence odds ratio (a-OR) and adjusted intensity ratio (a-IR) for two final models.

Variable	Number of fleas per host				Number of floor fleas per household			
	a-OR (95%CI) a	LR-test e	a-IR (95%CI) b	LR-test e	a-OR (95%CI) c	LR-test e	a-IR (95%CI) d	LR-test e
**Village level:**								
Topography of village						<0.001		
Mountain					Ref f			
Basin among mountains					0.42 (0.27–0.66)			
Number of households								0.005
≤80							Ref	
>80							3.21 (1.39–7.39)	
Houses raising chicken						0.002		0.013
≤80%					Ref		Ref	
>80%					2.86 (1.38–5.90)		11.59 (1.82–74.02)	
**Household level:**								
House construction						0.020		0.045
Earth and wood					Ref		Ref	
Brick and wood					0.39 (0.16–0.94)		0.09 (0.01–0.78)	
Host species		<0.001						
*S. murinus*	Ref							
*R. flavipectus*	10.00 (3.86–25.93)							
Seeing SM faeces in house		0.004						
No	Ref							
Yes	2.94 (1.37–6.31)							
Keeping dog						0.003		
No					Ref			
Yes					1.96 (1.25–3.06)			
Keeping cattle								0.025
No							Ref	
Yes							2.53 (1.11–5.76)	
Surrounding-house		0.003				0.042		
No	Ref				Ref			
Yes	7.43 (1.81–30.48)				2.20 (0.97–5.01)			
Paddy grown near house				0.050				
No			Ref					
Yes			0.47 (0.23–0.98)					
Location of toilet				0.009				
No toilet			Ref					
Inside toilet			0.45 (0.13–1.53)					
Outside toilet			2.25 (1.21–4.19)					

a Predicting whether the SM was infested.

b Predicting the mean number of fleas on any infested SM.

c Predicting whether the house was infested.

d Predicting the mean number of fleas in any infested house.

e p value from likelihood ratio test.

f Reference category.

At the village level, location of a village in the mountains increased the prevalence odds of household infestation with floor fleas, while larger size of village (>80 households) increased the intensity of infestation. Villages in which more than 80% of houses raising chicken were associated with increased prevalence odds and increased intensity of household floor flea infestation. At the household level, house constructed with earth and wood were associated with increased prevalence odds and intensity of household floor flea infestation. Locations in areas with adjacent houses and keeping dog were associated with increased prevalence odds of infestation. Keeping cattle was associated with increased intensity of infestation.

## Discussion

In this investigation, two species, *X. cheopis* and *L. segnis*, were collected from 101 of 166 SMs. The flea prevalence and flea abundance of *R. flavipectus* were higher than those of *S. murinus*. There was no association between the prevalence of floor flea and of SM/host flea in houses. Household-level variables influenced the abundance of host flea and floor flea, while village-level variables influenced only the abundance of floor flea.

Among the 4 flea species collected, *X. cheopis* is of great public health significance because it is the primary vector of bubonic plague, particularly in commensal rodent plague foci [6]. This was the most common species found on hosts and the second most common on the floor in this study. Therefore, the risk of plague occurrence cannot be excluded in these endemic villages. *P. irritans* (the human flea) has also been reported to be an important vector of human plague in Yunnan province [21]. Previous studies in Yunnan, Guangxi and Hebei province of China reported that this species was the predominant species accounting for 61% to 99% of all floor fleas [7]; [11]; [22]. In contrast, only 6 human fleas (1.9%) were collected from floors in this study. Perhaps the different location or seasonal fluctuation are responsible for this difference. *C. felis felis* (a subspecies of cat flea) is also able to transmit plague to humans from pets [23], while *L. segnis* (mouse flea) is believed to be a weak vector or unable to transmit plague [21]; [23]. It should be noted that *C. felis felis* was the dominant species among floor fleas, accounting for 65.1% in the present study.

The lack of any correlation between the number of *L. segnis* and the number of *X. cheopis* on *R. flavipectus* argues against a facilitating or competitive relationship between these two species, but supports the concept of separate niches on the hosts. Previous studies have reported that certain host species present better habitats for multiple flea species [24]; [25]. The coexistence of flea species is related both to the structure of flea communities and the affinities of host species [26]; [27]. In contrast, the positive, though weak, correlation at the household level between the number of *X. cheopis* fleas and the number of *C. felis felis* fleas on the floor implies that a relationship may exist in the off-host environment. The relationship may be caused by environmental (such as house hygiene conditions) or host-associated blood factors that make certain house more suitable for flea infestation.

The flea abundance on *R. flavipectus* was higher than that on *S. murinus*. This was a result of a difference in flea prevalence, rather than in flea intensity, which was not shown to differ between the two species. Previous studies have reported that host species, as well as body size, weight and age, affect flea infestation on the host. Species of larger body size have higher flea prevalence and abundance [28]–[30]. It was explained that larger hosts have greater carrying capacities than smaller hosts of the same or different species [28]. Unfortunately, these host parameters were not measured in this study.

Both *L. segnis* and *X. cheopis* infested *R. flavipectus*, while *S. murinus* was infested only by *X. cheopis*. Both the flea prevalence and abundance were significantly higher on *R. flavipectus* than on *S. murinus*. These differences between the two host species suggest that fleas preferred to infest *R. flavipectus* (belonging to Rodentia) over *S. murinus* (belonging to Soricomorpha). Previous studies reported that many SMs that share the same habitat niches also share flea species, but there is a great variance in the host specificity or preference [29]; [31]; [32]. In Brazil, among 12 orders of mammals found to be parasitized, rodents were the preferred hosts [33].

A flea is able to relocate from one host to another via social interaction between hosts, when a host visits an alien burrow, and when a flea leaving its host and dispersing freely [34]–[36]. The flea transmission rates among hosts mainly rely on host population density in natural parasite communities [37]. This is consistent with the findings that the closeness of houses was associated with increased SM abundance [14]. SM abundance was indicated in our study by seeing SM faeces, which showed statistically significant association with the host flea prevalence.

The movement of hosts seeking food or mating is quite common. During host-to-host transfer, environmental conditions greatly affect hosts as well as their ectoparasites [29]; [38]. The reason of the effect of two household-level environment variables, namely the lack of paddy grown near house and the outside location of toilet appeared to increase host flea intensity is not clear. However, the latter factor has some public health implication. Outside toilets in the study areas are usually of an open type. They are known to facilitate the transmission of several food- and water-borne diseases and increase the population of pests. Our data further emphasize that this type of toilet is associated with increased flea intensity on their small-mammal hosts.

Most studies have estimated flea numbers by relying exclusively on sampling from the host body [39]–[43]. However, floor fleas have been shown to harbour *Yersinia pestis* in a plague outbreak in Yunnan province [44]. Our results showed that floor fleas accounted for about half of total fleas (315 out of 659) captured in houses. Apart from underestimating household flea population to which humans are likely to be exposed, a lack of floor flea data may lead to incomplete understanding of plague ecology. Therefore, sampling from both the host body and the off-host environment (such as floors) may improve the accuracy of estimating flea abundance.

In this study, the composition of floor and host flea species was quite different. There was no apparent association between the total numbers of floor fleas and host fleas at either village or household level. These features imply that the SMs might not be the main source of the floor fleas. In USA, Egypt, Libya, and Europe, *C. felis felis* is the predominant flea specie found on dogs and cats [45]–[48]. This flea species is also capable of infesting livestock including horses [49], goats [50]; [51] and cattle [52]; [53]. In this investigation, about one third of households raised guard dog and 41% of households raised bovine to help with farming tasks. Perhaps this could explain the large proportion (65.1%) of *C. felis felis* among floor fleas. Keeping a dog in the house increased the floor flea prevalence, keeping cattle increased the floor flea intensity, but, surprisingly, there was no evidence in this study that floor flea prevalence was associated with keeping cats.

About two thirds of households as well as >80% of houses at the village-level raise chickens, this practice increased not only the floor flea prevalence (OR  =  2.9) but also the floor flea intensity (IR  =  11.6). This suggests that keeping chicken was a risk factor for flea infestation on the floor. However, few studies have reported such association between flea infestation and keeping chicken. Okaeme (1988) reported that *C. felis felis* infested domestic chicken in Nigeria [54] and Rahbari et al. (2008) reported that chickens infested by three flea species including *P. irritans*, *C. canis* and *C. gallinae* in Iran but the flea prevalence of chicken was lower than that of cattle and goat [41]. Unfortunately, we did not collect data on flea infestation of these domestic animals.

Higher floor flea prevalence was associated with the location of houses adjacent to other houses and higher floor flea intensity was associated with villages having a larger number of houses (>80 households). This suggests that floor fleas can transfer from house to house. It is known that individual flea can disperse rather long distances by host [35]. But the means of the transfer of floor fleas, either independently or on their hosts, or both, are unclear. In addition, lower prevalence and intensity of floor fleas were found in houses constructed with brick and wood. This may be related to the hygiene conditions. Although the general quality of sanitation was not recorded, investigators observed that the sanitation of brick and wood houses was generally better than that of the earth and wood houses.

Ambient temperature and relative humidity greatly affect the abundance of fleas via their influence on survival [12]; [13]. The lower prevalence of floor fleas in villages located in basin areas than that in mountain areas might be explained by differences in climate. Valley areas may have relatively higher temperatures therefore adversely affect the survival of fleas. Further studies are needed to confirm this.

In contrast to most previous reports on the host flea ecology, the current paper added potential importance of floor fleas which have been scarcely looked at. The nature of floor flea reported in this study is still incomplete. Further studies are needed.

In conclusion, there was no evidence of association between floor flea and host flea in the same house. Flea populations on hosts and on floors are influenced by several ecological and hygiene factors. This means that rodent control alone may not be sufficient to control plague in these areas. Plague control programs should also pay attention to ecological and hygiene factors in order to have successful results.

## Supporting Information

Alternative Language Abstract S1 Translation of Abstract into Chinese by Jia-Xiang Yin.
(0.03 MB DOC)
