# Supplementary material for: Predictors for Abundance of Host Flea and Floor Flea in Households of Villages with Endemic Commensal Rodent Plague, Yunnan Province, China
Source: PLoS Negl Trop Dis. 2011 Mar 29;5(3):e997. doi: 10.1371/journal.pntd.0000997 (PMC3066137; doi:10.1371/journal.pntd.0000997)
Supplement: Alternative Language Abstract S1 — Translation of Abstract into Chinese by Jia-Xiang Yin. (0.03 MB DOC) [file pntd.0000997.s001.doc]

**云南省家鼠鼠疫疫源地室内鼠体寄生蚤和地面游离蚤丰盛度预测因子的研究**

尹家祥^1^ Alan Geater^2^ Virasakdi Chongsuvivatwong^2^ 董兴齐^1^ 杜春红^1^ 钟佑宏^1^

^1^ 云南省地方病防治所 云南省大理市文化路5号 671000

^2^ 泰国宋卡王子大学医学院流行病学教研室 泰国宋卡府合艾市 90112

**前言** 1990年至2006年间，云南省梁河县55个自然村至少发生过一次鼠疫流行。该研究的目的是调查梁河县鼠疫疫源村室内鼠体寄生蚤和地面游离蚤丰盛度以及确定影响丰盛度的因子。**方法** 通过捕获宿主动物和布放粘蚤纸收集跳蚤，使用调查问卷并采用访谈方式收集人口统计学资料、环境因素和其它相关信息。应用多元跨栏负二项分布模型确定影响跳蚤丰盛度的因子。**结果** 101只小型哺乳动物（94只黄胸鼠和7只臭鼩鼱）体表上收集到344匹跳蚤。黄胸鼠跳蚤感染率和丰盛度高于臭鼩鼱，但两者的寄生蚤密度没有显著差异。104个家庭收集到共计305匹地面游离蚤。印鼠客蚤和猫栉首蚤指明亚种分别是鼠体寄生蚤和地面游离蚤的优势种。发现鼠粪和捕获黄胸鼠的家庭分别增加小型哺乳动物跳蚤感染率2.9倍和10倍。养狗家庭增加2倍的地面游离蚤孳生概率。养牛家庭增加153％的地面游离蚤密度。80％以上家庭养鸡的自然村增加地面游离蚤孳生概率和蚤密度分别是2.9倍和11.6倍。砖木结构和土木结构的房屋相比，地面游离蚤孳生概率和蚤密度分别降低60％和90％。周围有其他住户的家庭，其鼠体寄生蚤和地面游离蚤的孳生概率分别增加7.4倍和2.2倍。被调查家庭周围有水稻生长，其鼠体寄生蚤密度降低53％，而厕所位于房屋外的家庭鼠体寄生蚤密度增加125％。**结论** 在家鼠鼠疫疫源地，单一灭鼠难以达到预防和控制鼠疫感染的目的。为了成功防控鼠疫，除灭鼠外，尚需重视影响跳蚤孳生的生态环境和卫生状况等因素。

**关键词：**动物鼠疫疫源地；鼠体寄生蚤；地面游离蚤；丰盛度；预测因子；云南省；中国

Translation of the abstract into Chinese was made by the first author Jia-Xiang Yin.
